# Draft Genome Sequences of the Type Strains of Actinobacillus indolicus (46K2C) and Actinobacillus porcinus (NM319), Two NAD-Dependent Bacterial Species Found in the Respiratory Tract of Pigs

**DOI:** 10.1128/MRA.00716-19

**Published:** 2020-01-02

**Authors:** Janine T. Bossé, Yanwen Li, Roberto Fernandez Crespo, Øystein Angen, Matthew T. G. Holden, Lucy A. Weinert, Duncan J. Maskell, Alexander W. Tucker, Brendan W. Wren, Andrew N. Rycroft, Paul R. Langford

**Affiliations:** a Section of Paediatric Infectious Disease, Department of Infectious Disease, Imperial College London, London, United Kingdom; b Department of Microbiology and Infection Control, Statens Serum Institut, Copenhagen, Denmark; c The Wellcome Trust Sanger Institute, Cambridge, United Kingdom; d Department of Veterinary Medicine, University of Cambridge, Cambridge, United Kingdom; e Faculty of Infectious & Tropical Diseases, London School of Hygiene & Tropical Medicine, London, United Kingdom; f Department of Pathology and Pathogen Biology, The Royal Veterinary College, Hatfield, United Kingdom; University of Rochester School of Medicine and Dentistry

## Abstract

We report here the draft genome sequences of the type strains of Actinobacillus indolicus (46K2C) and Actinobacillus porcinus (NM319). These NAD-dependent bacterial species are frequently found in the upper respiratory tract of pigs and are occasionally associated with lung pathology.

## ANNOUNCEMENT

Bacteria belonging to the family *Pasteurellaceae* that inhabit the porcine respiratory tract and require NAD (also called the V factor) for growth include Actinobacillus pleuropneumoniae, Glaesserella parasuis (formerly Haemophilus parasuis), Actinobacillus indolicus, Actinobacillus porcinus, Actinobacillus minor, and a separate species tentatively designated “Actinobacillus porcitonsillarum,” which, although phenotypically more similar to A. pleuropneumoniae, is phylogenetically closer to *A. minor* (1–5). The first two species are important pathogens, whereas the latter four are considered commensals of the upper respiratory tract (1, 3, 6), though isolates of *A. indolicus* and *A. porcinus*, associated with lung pathology, have been identified (1). For A. pleuropneumoniae, there are two biotypes based on the requirement for NAD, with most isolates belonging to the NAD-dependent biovar 1 (7, 8).

The phylogeny of the *Pasteurellaceae* is complicated, and there remain numerous species with uncertain taxonomic status (4, 9, 10). Whole-genome sequences, which can help clarify evolutionary relationships and the development of differential diagnostics, are available for all of the above-mentioned bacteria except *A. indolicus* and *A. porcinus*. We therefore chose to sequence the type strains, 46K2C and NM319, respectively, of these species. Both 46K2C^T^ and NM319^T^ were originally isolated from the upper respiratory tract of pigs in Denmark (11) and Canada (12), respectively. They were initially identified as *Haemophilus* taxons D and F, respectively, prior to their designation as the type strains of the newly assigned species in 1996 (1, 2).

Strains 46K2C^T^ and NM319^T^ were obtained from the Culture Collection University of Gothenburg (CCUG 39029^T^ and CCUG 38924^T^, respectively), stored frozen at −80°C in 25% glycerol, and minimally passaged (up to four times) on brain heart infusion agar (Difco) supplemented with 0.01% NAD prior to genomic DNA extraction using a FastDNA spin kit (MP Biomedicals), according to the manufacturer’s protocol for bacterial cells. Paired-end libraries were prepared from 500 ng of genomic DNA, as previously described (13), using the modified Illumina protocol developed by Quail et al. (14) for sequencing at the Wellcome Sanger Institute (Hinxton, UK) on an Illumina HiSeq 2000 analyzer for 75 cycles. Draft genome sequences were assembled *de novo* using a previously reported pipeline (15). Briefly, the 2 × 76-bp paired reads were used to create multiple assemblies for each species’ genome using Velvet v1.2 (16) and VelvetOptimiser v2.2.5 (http://bioinformatics.net.au/software.velvetoptimiser.shtml). For each genome, the assembly with the best *N*_50_ value was further improved by scaffolding contigs using SSPACE (17), with any gaps filled by GapFiller (18). Automated annotation was performed for both genome assemblies using Prokka v1.5 (19). Default parameters were used for all software programs.

The draft genome of *A. indolicus* 46K2C^T^ was assembled from 2,031,716 total reads, with an average quality score of 37.9, into 22 contigs (*N*_50_, 183,245; *L*_50_, 4), with a total length of 2,103,350 bases. This sequence encodes 1,956 predicted proteins and has a G+C content of 40.1%, which is higher than the 35.5% originally determined (2) but lower than the value of 42.6% calculated by Kuhnert and Korczak based on the G+C content of selected genes (20). The draft genome of *A. porcinus* NM319^T^ was assembled from 1,733,818 reads, with an average quality score of 37.8, into 29 contigs (*N*_50_, 182,117; *L*_50_, 5), with a total length of 2,280,774 bases. This sequence encodes 2,095 predicted proteins and has a G+C content of 41.1%, which is similar to values (41.4% and 41.6%) previously reported for this strain (2, 20).

The availability of the draft genome sequences of the type strains of *A. indolicus* and *A. porcinus* will facilitate differentiation of these species from other NAD-dependent bacteria found in the porcine respiratory tract and contribute to the understanding of *Pasteurellaceae* phylogeny and host interactive biology.

### Data availability.

The draft genome assemblies for *A. indolicus* 46K2C^T^ and *A. porcinus* NM319^T^ have been deposited in the European Nucleotide Archive (ENA) under BioProject number PRJEB31492. Raw sequence reads have been deposited in the ENA under accession numbers ERS134282 and ERS134597, respectively. The ENA accession numbers for the assembled contigs are GCA_901764975 (CABFKH010000001 through CABFKH010000022) and GCA_901764995 (CAAGST010000001 through CAAGST010000029), respectively.
